# Effect of Smoking Reduction Therapy on Smoking Cessation for Smokers without an Intention to Quit: An Updated Systematic Review and Meta-Analysis of Randomized Controlled Trials

**DOI:** 10.3390/ijerph120910235

**Published:** 2015-08-25

**Authors:** Lei Wu, Samio Sun, Yao He, Jing Zeng

**Affiliations:** 1 Department of Epidemiology, Institute of Geriatrics, Beijing Key Laboratory of Aging and Geriatrics, Chinese People’s Liberation Army General Hospital, Beijing 100853, China; E-Mails: wlyg0118@163.com (L.W.); jingzeng1991@126.com (J.Z.); 2 Department of Bioengineering, The University of Tokyo, 1138656, Japan; E-Mail: samio4762@gmail.com; 3 State Key Laboratory of Kidney Disease, Chinese People’s Liberation Army General Hospital, Beijing 100853, China

**Keywords:** smoking reduction therapy, without quit intention, varenicline, nicotine replacement therapy, meta-analysis

## Abstract

*Objective*: Effective strategies are needed to encourage smoking cessation for smokers without an intention to quit. We systematically reviewed the literature to investigate whether smoking reduction therapy can increase the long-term cessation rates of smokers without an intention to quit. *Methods*: PubMed, Embase, and CENTRAL (Cochrane Central Register of Controlled Trials) were searched for randomized controlled trials (RCTs) on the effect of smoking reduction therapy on long-term smoking cessation in smokers without an intention to quit. The primary outcome was the cessation rate at the longest follow-up period. A random effects model was used to calculate pooled relative risks (RRs) and 95% confidence intervals (CIs). *Results*: Fourteen trials with a total of 7981 smokers were included. The pooled analysis suggested that reduction support plus medication significantly increased the long-term cessation of smokers without an intention to quit compared to reduction support plus placebo (RR, 1.97; 95% CI, 1.44–2.7; I^2^, 52%) or no intervention (RR, 1.93; 95% CI, 1.41–2.64; I^2^, 46%). In a subgroup of smokers who received varenicline or nicotine replacement therapy (NRT), the differences were also statistically significant. This suggests the safety of using NRT. The percentage of smokers with serious adverse events who discontinued because of these events in the non-NRT group was slightly significantly different than in the control group. Insufficient evidence is available to test the efficacy of reduction behavioural support in promoting long-term cessation among this population. *Conclusions*: The present meta-analysis indicated the efficacy of NRT- and varenicline-assisted reduction to achieve complete cessation among smokers without an intention to quit. Further evidence is needed to assess the efficacy and safety of reduction behavioural support and bupropion.

## 1. Introduction

Smoking is still one of the largest preventable causes of death in the world. Although smoking cessation can reduce the chance of developing smoking-related diseases, 75.6% of Chinese smokers have no plan to quit smoking [1]. A similar phenomenon has been observed in other developed countries [2,3]. These results reveal the necessity for an effective strategy to encourage cessation in smokers without an intention to quit.

Smoking reduction therapy might increase the likelihood of complete cessation, and it has been recommended as a therapeutic choice for cigarette smokers without an intention to quit [4,5]. One of the biggest uncertainties with this method is the association between short-term reduction of daily cigarette consumption and long-term complete cessation. There has been some concern that future attempts to quit smoking might be undermined by short-term reduction; others have argued that the reduction might be an intermediate step before quitting completely [6].

Several reviews have assessed the efficacy of methods for helping smokers without an intention to quit [7,8,9,10,11]. Differences between the current meta-analysis and previous meta-analyses on the same topic should be noted. A meta-analysis by Hughes *et al*. included studies with different designs (such as cross-sectional, prospective studies and RCTs) [7]. Due to the heterogeneity of the methods and results, a qualitative review was performed instead of a meta-analysis [7]. Two meta-analyses examined only NRT-aided reduction [8,9]. Recently, several studies on this topic have advocated for the use of varenicline to reduce the daily consumption of cigarettes to achieve the goal of complete cessation. A Cochrane review by Stead *et al*. included smokers who were willing to quit smoking [10]. In a meta-analysis focusing on the same population (unwilling to quit smoking), Asfar *et al*. only included self-reported point prevalence of cessation at the end of follow-up. In addition, they did not consider the safety of using smoking cessation medication as an aid in reduction [11].

Considering the accumulating evidence, we conducted an updated systematic review and meta-analysis to assess the efficacy and safety of smoking reduction therapy for smokers without an intention to quit. In contrast with the above-discussed meta-analyses, we grouped samples into four categories according to different types of smoking reduction therapies. We also restricted our analysis to only randomized, controlled clinical trials and used the strictest available criteria (sustained, biochemically validated and long follow-up). Moreover, we widely considered different categories according to different types of smoking reduction therapy.

## 2. Methods

### 2.1. Literature Search

This is a systematic review and meta-analysis of previously published randomized controlled trials (RCTs). We conducted and reported the current study in adherence with Preferred Reporting Items for Systematic Reviews and Meta-Analyses (PRISMA) [12]. We searched PubMed, Embase, and Cochrane Central Register of Controlled Trials (CENTRAL) for records to report the effect of smoking reduction therapy on smokers with no intention to quit. The search terms included “tobacco reduction”, “cigarette * reduction”, “reduce smoking”, “smoking reduction”, “unwilling to”, “not willing”, “no inten *”, “not ready”, “not interest *”, “uninterest *”, and “unmotivated”. Details of the search strategy are shown in Supplementary 1. No language and data restriction were imposed. The last search was run on 24 April 2015. We manually searched the reference lists of relevant studies to identify other potentially eligible studies.

### 2.2. Selection Criteria

Two investigators (Lei Wu and Samio Sun) independently performed the initial search. Duplicate records were deleted; the titles and abstracts of each trial were screened. We identified each study as excluded or requiring further assessment.

We included studies that met the following criteria: (1) population: adult smokers who were not ready to quit, were unwilling to quit or had no intention to quit smoking (willing or unwilling to reduce their smoking intensity); (2) intervention: smoking cessation medications to assist with smoking reduction (such as gum, inhalable nicotine replacement therapy, varenicline or bupropion) or behavioural support/the provision of self-help materials to promote reduction; (3) comparison: placebo, no intervention, and other behavioural support (other support for smoking cessation with the exception of reduction support); (4) outcome: abstinence from smoking after at least six months of follow-up; and (5) design: randomized controlled trials (RCTs).

### 2.3. Data Extraction

Lei Wu and Samio Sun independently performed data extraction. The following data were extracted from each study: first author, date and place of publication, patient characteristics, number of patients enrolled (each arm and total), summary of intervention and control conditions, reported outcomes and risk of bias. The extracted data were entered into a standardized Excel (Microsoft Corporation, Seattle, WA, USA) file. Any disagreements were resolved by discussion between the two investigators. If a trial had multiple arms, we reused the control group in each comparison.

The primary outcome was the smoking cessation rate at the longest follow-up period (at least six months from the baseline intervention). In each trial, the strictest available criteria (sustained, biochemically validated, and longest follow-up) were used to define the quit rate. We collected data measured during an intention-to-treat analysis. The secondary outcome included serious and non-serious adverse events that were reported in the included trials.

### 2.4. Quality Assessment

The Cochrane Collaboration tool was used to assess the risk of bias in each trial [13]. A value of “high”, “low”, or “unclear” risk of bias was assigned according to the following domains: random sequence generation (selection bias), allocation concealment (selection bias), blinding of participants and personnel (performance bias), blinding of outcome assessment (detection bias), incomplete outcome data (attrition bias), selective reporting (reporting bias) and other biases. Disagreements were resolved by discussion with a third author (Yao He). 

### 2.5. Data Synthesis 

We calculated the relative risks (RRs) with 95% confidence intervals (CIs) for the dichotomous outcome data. Because the sample size, population characteristics, and other confounding factors were not consistent among studies, a random effects model was used to pool the outcome data, regardless of heterogeneity [14]. Heterogeneity was assessed using the I^2^ statistic. Studies with an I^2^ statistic >50% were indicated to have significant heterogeneity [15]. We further performed subgroup analysis according to the type of smoking cessation medication (nicotine replacement therapy, varenicline and bupropion). The influence of a single study on the overall pooled results was estimated by omitting one study at every turn. Sensitivity analyses were performed to explore the influence of various exclusion criteria on the overall pooled estimate.

The presence of publication bias was evaluated by using the Begg and Egger tests [16,17]. Results were considered as statistically significant for *p* value <0.05. We used Stata (version 12.0; StataCorp LP, College Station, Texas, USA) and Review Manager Software (version 5.2; The Nordic Cochrane Centre, Copenhagen, Denmark) for the statistical analyses.

## 3. Results

### 3.1. Study Identification and Selection

**Figure 1:**
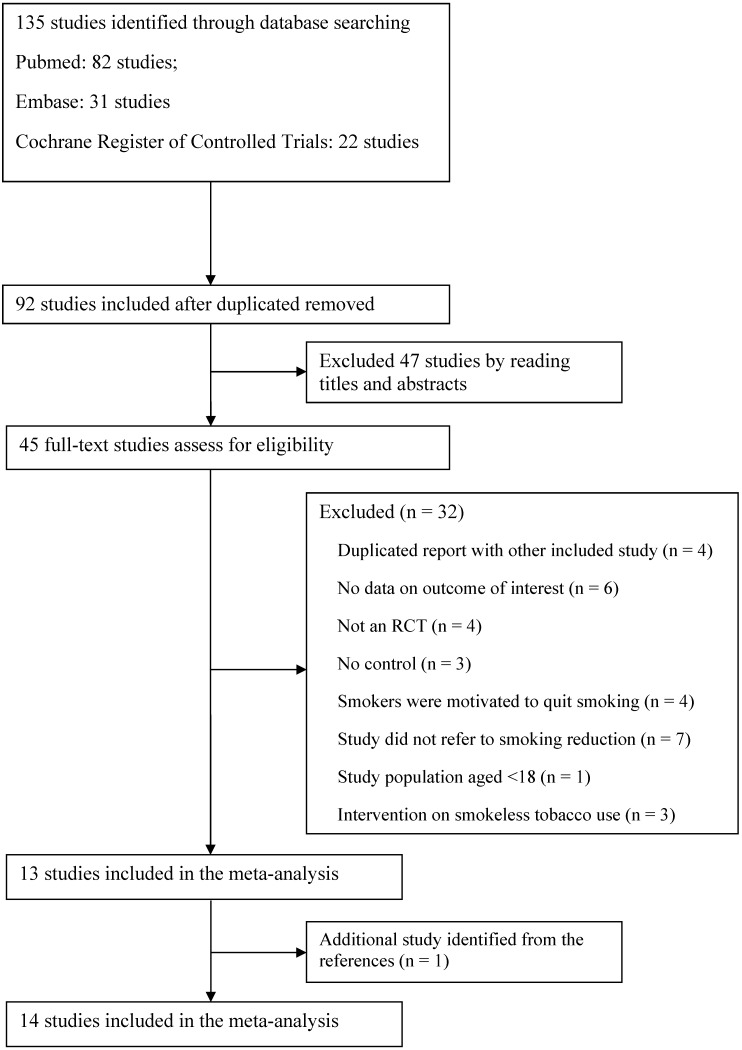
Flow diagram of trials included in the meta-analysis.

A detailed flow diagram of the trials included in the meta-analysis is shown in Figure 1. A total of 135 records were identified from the initial database search. Of these, 92 records were excluded for duplicates, and 47 records were excluded after reading the titles and abstracts. The remaining 45 full-text articles were assessed for eligibility. An additional study was identified from the references. Finally, 14 studies were included in the present meta-analysis [18,19,20,21,22,23,24,25,26,27,28,29,30,31].

### 3.2. Study Characteristics

Table 1 shows the main characteristics of the included trials, and Table 2 lists the outcome data of each included trial. These trials were published between 2000 and 2015. Seven of the included trials were conducted in the United States. The sample size ranged from 67 to 1410 (total 7981). The follow-up period ranged from 6 months to 60 months.

Ten of the included trials reported biochemically validated quit rates (confirmed by exhaled carbon monoxide). Among the primary outcomes, 7-day time point and continuous abstinence rates were reported in 10 and four trials, respectively. Among the secondary outcomes, adverse events were reported in 11 trials.

The studies were grouped into four comparisons according to the following different types of smoking reduction therapy (Table 2): (a) reduction support (in the current study, reduction support was defined as behavioural interventions or self-help materials to increase reduction; smoking cessation medications were not incorporated in this definition) plus medication versus reduction support plus placebo, (b) reduction support plus medication versus no intervention, (c) reduction support plus medication versus other support plus medication, and (d) reduction support versus no intervention. Four trials had three arms [21,22,23,24]. We reused the above arm matching for each comparison (e.g., a trial by Carpenter, *et al*. had the following three arms: (1) telephone-based reduction support plus NRT plus brief advice group, (2) motivational advice plus NRT for quit attempt plus brief advice group, and (3) a group with no intervention. For consistency, we analysed the differences in the cessation rates between (1) and (2) and between (1) and (3).)

### 3.3. Quality Assessment

Supplementary Figure S1 summarizes the details of the risk-of-bias assessment. Eight trials had a detailed description of random sequence generation, and five trials reported appropriate allocation concealment. Five trials reported that the participants and personnel were blinded to the nature of the examined products (medication or placebo). All trials were judged to have low risks of incomplete outcome data, reporting bias and other bias.

### 3.4. Primary Outcome

#### 3.4.1. Reduction Support Plus Medication *vs.* Reduction Support Plus Placebo

**Figure 2:**
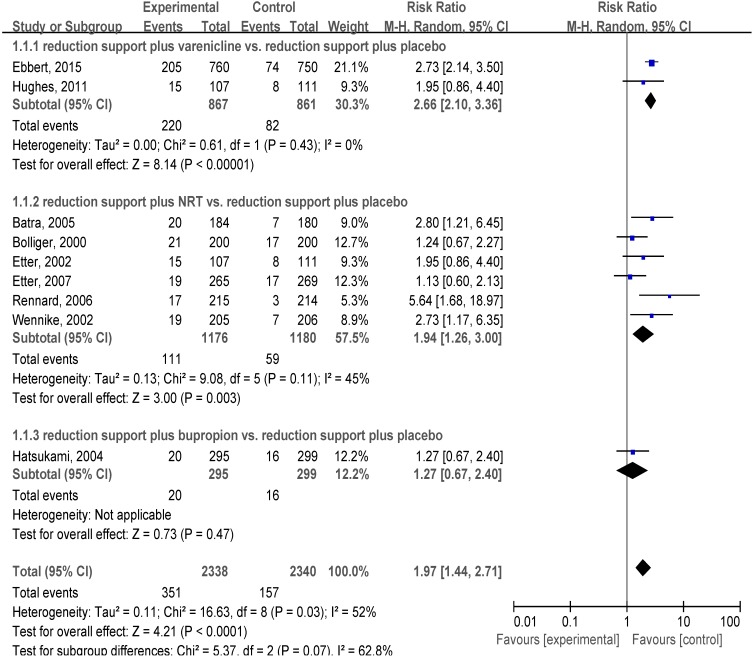
Forest plot of comparison: reduction support plus medication *vs.* reduction support plus placebo.

We estimated the pooled effect size of reduction support plus medication versus reduction support plus placebo based on nine trials (Figure 2). The pooled relative risk (RR) was 1.97, and the 95% confidence interval (CI) was 1.44 to 2.71, with evidence of significant heterogeneity (I^2^ = 52%). In the subgroup analysis, two of the nine trials were offered varenicline or placebo for smokers with no intention to quit, and the pooled RR was 2.66 and 95% CI was 2.10 to 3.36 with no evidence of significant heterogeneity (I^2^ = 0%). Six of the nine trials offered nicotine replacement treatment (NRT) or placebo, and the pooled RR was 1.94 and 95% CI was 1.26 to 3.00 with no evidence of significant heterogeneity (I^2^ = 45%). One study offered bupropion or placebo (RR, 1.27; 95% CI, 0.67–2.40). Two trials offered self-help materials rather than behavioural reduction support to assist in reduction [23,24].

#### 3.4.2. Reduction Support Plus Medication *vs.* no Intervention

**Figure 3:**
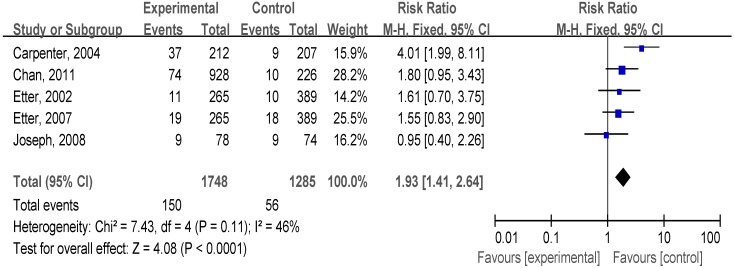
Forest plot of comparison: reduction support plus medication *vs.* no intervention.

We included five trials to test the efficacy of reduction support combined with smoking cessation medication. As shown in Figure 3, compared with no intervention, those smokers who received reduction support plus medication had significantly increased smoking abstinence (RR, 1.93; 95% CI, 1.41–2.64; I^2^ = 46%).

**Table 1:** Characteristics of the included trials.

Trial	Sample Size	Design	Setting	Initial Intention to Quit	Population	Male N (%)		Age (Years) Mean (SD)		Cigarettes/Day Mean (SD)	
						Treat Control		Treat Control		Treat Control	
Bolliger, 2000 [18]	400	multi-centre	Switzerland	willing to reduce their smoking but unable or unwilling to stop smoking immediately	healthy	104 (52.0)	86 (43.0)	45.8 (10.5)	46.4 (10.5)	30.3 (12.1)	28.2 (11.4)
Batra, 2005 [19]	364	multi-centre	Switzerland	willing to change their smoking behavior but unwilling to quit	healthy	101 (54.1)	117 (64.8)	42.6 (9.9)	43.5 (10.3)	27.9 (9.2)	29.6 (9.5)
Carpenter, 2003 [20]	67	single-centre	United States	no interest in quitting smoking in the next 30 days	healthy	26 (74.3)	20 (62.5)	43 (12)	44 (9)	24 (10)	23 (10)
Carpenter, 2004 [21]	616	single-centre	United States	did not wish to quit	healthy	144 (67.9)	123 (62.4) 139 (67.1)	38 (12)	39 (13) 41 (14)	23 (10)	21 (8) 22 (9)
Chan, 2011 [22]	1154	single-centre	Hong Kong	no intention to quit in the near future but interested in reducing smoking	healthy	748 (80.6)	198 (87.6)	41.9 (10.3)	42.5 (11.2)	19.9 (9.8)	19.2 (8.9)
Etter, 2002 [23]	923	single-centre	Switzerland	no intention to quit smoking in the next 6 months	healthy	143 (54.0)	132 (49.0) 171 (44.0)	43.2	41.7 42.9	29.8 (10.3)	29.4 (9.4) 30.2 (10.4)
Etter, 2007 [24]	923	single-centre	Switzerland	no intention of quitting smoking in the next 6 months	healthy	54 (20.4)	49 (18.2) 44 (11.3)	43.2	41.7 42.9	29.8 (10.3)	29.4 (9.4) 30.2 (10.4)
Ebbert, 2015 [25]	1410	multi-centre	10 countries	not willing or able to quit smoking within the next month but willing to reduce smoking and make a quit attempt within the next 3 months	healthy	425 (55.9)	426 (56.8)	44.7 (11.8)	44.4 (12.0)	20.6 (8.5)	20.8 (8.2)
Glasgow, 2009 [26]	320	single-centre	United States	not interested in quitting smoking at that time	healthy	44 (26.8)	44 (28.2)	54.8 (10.4)	56.0 (11.3)	21.2 (9.4)	20.1 (9.0)
Hatsukami, 2004 [27]	594	multi-centre	United States	motivated to reduce their cigarette usage, but who were unwilling or perceived themselves to be unable to quit smoking at the time of screening	healthy	169 (57.3)	158 (52.8)	42.5 (11.0)	42.0 (11.6)	29.0 (9.8)	28.5 (9.6)
Hughes, 2011 [28]	218	multi-centre	United States	interested in quitting but had no plans to quit in the next month	healthy	65 (60.7)	63 (56.8)	44 (14)	41 (15)	19 (9)	17 (7)
Joseph, 2008 [29]	152	multi-centre	United States	unwilling or uninterested in setting a stop smoking date in the next 30 days	cardiovascular patient	70 (89.7)	65 (87.8)	57.5 (8.6)	58.4 (9.6)	27.7 (12.5)	27.0 (11.0)
Rennard, 2006 [30]	429	multi-centre	United States	did not plan to quit smoking within the next 4 weeks, but want to reduce cigarette consumption	healthy	88 (40.9)	104 (48.6)	45.9 (12.3)	44.8 (12.1)	29.3 (10.1)	30.4 (9.9)
Wennike, 2002 [31]	411	single-centre	Denmark	unwilling or unable to quit smoking, but interested in reducing their smoking	healthy	72 (35.0)	85 (41.0)	45 (10)	44 (10)	24 (7)	24 (7)

SD, standard deviation.

**Table 2:** Outcome data of the included trials.

Trial	Treat *vs.* Control	Treatment Duration (Months)	Follow-up (Months)	Carbon Monoxide-Confirmed	Outcome	Quit Rate	
						Treat/Total N (%)	Control/Total N (%)
**Reduction support plus medication versus reduction support plus placebo**							
Bolliger, 2000 [18]	nicotine inhaler plus reduction counseling *vs.* placebo inhaler plus reduction counseling	18	24	Yes	7-day point	21/200 (10.5)	17/200 (8.5)
Batra, 2005 [19]	4-mg nicotine gum plus reduction counseling *vs.* placebo gum plus reduction counseling	12	13	Yes	7-day point	20/184 (10.9)	7/180 (3.9)
Etter, 2002 [23]	NRT (gum, inhaler or patch) plus reduction booklet *vs.* placebo plus reduction booklet	6	6	No	sustained for 4 weeks	11/265 (4.2)	5/269 (1.9)
Etter, 2007 [24]	NRT (gum, inhaler or patch) plus self-help reduction material *vs.* matching placebo plus self-help reduction material	6	60	No	sustained for 60 months	19/265 (7.2)	17/269 (6.3)
Ebbert, 2015 [25]	Varenicline plus four brief reduction counseling *vs.* placebo plus four brief reduction counseling	6	12	Yes	sustained (week 21 to 52)	205/760 (27.0)	74/750 (9.9)
Hatsukami, 2004 [27]	bupropion plus reduction counseling *vs.* placebo plus reduction counseling	12	12	Yes	sustained (week 4 to 26)	20/295 (6.8)	16/299 (5.4)
Hughes, 2011 [28]	Varenicline plus four brief reduction counseling *vs.* placebo plus four brief reduction counseling	2 (4)	6	Yes	7-day point	15/107 (14.0)	8/111 (7.2)
Rennard, 2006 [30]	10-mg nicotine inhaler plus reduction counseling *vs.* placebo inhaler plus reduction counseling	12	15	Yes	7-day point	17/215 (7.9)	3/214 (1.4)
Wennike, 2002 [31]	nicotine gum plus reduction counseling *vs.* placebo gum plus reduction counseling	12	24	Yes	7-day point	19/205 (9.3)	7/206 (3.4)
**Reduction support plus medication versus no intervention**							
Carpenter, 2004 [21]	telephone-based reduction counseling plus NRT (gum or patch) plus brief advice *vs.* no intervention	6	6	No	7-day point	37/212 (17.5)	9/207 (4.3)
Chan, 2011 [22]	NRT plus 4 brief reduction counseling *vs.* simple advices	6	6	Yes	7-day point	74/928 (8.0)	10/226 (4.4)
Etter, 2002 [23]	NRT (gum, inhaler or patch) plus reduction booklet *vs.* no intervention	−	−	−	−	11/265 (4.2)	10/389 (2.6
Etter, 2007 [24]	NRT (gum, inhaler or patch) plus reduction material *vs.* no treatment	−	−	−	−	19/265 (7.2)	18/389 (4.6)
Joseph, 2008 [29]	NRT (gum or patch) plus reduction counseling *vs.* no intervention	18	18	No	7-day point	9/78 (11.5)	9/74 (12.2)
**Reduction support plus medication versus other support plus medication**							
Carpenter, 2003 [20]	NRT (gum, inhaler or patch) plus reduction counseling *vs.* NRT plus brief advice	6	6	Yes	7-day point	6/35 (17.1)	3/32 (9.4)
Carpenter, 2004 [21]	telephone-based reduction counseling plus NRT (gum or patch) plus brief advice *vs.* motivational advice plus NRT for quit attempt plus brief advice	−	−	−	−	37/212 (17.5)	46/197 (23.4)
**Reduction support versus no intervention**							
Glasgow, 2009 [26]	behavioral reduction supports *vs.* no intervention	12	12	Yes	7-day point	11/164 (6.7)	7/156 (4.5)

#### 3.4.3. Reduction Support Plus Medication *vs.* other Support Plus Medication

**Figure 4:**
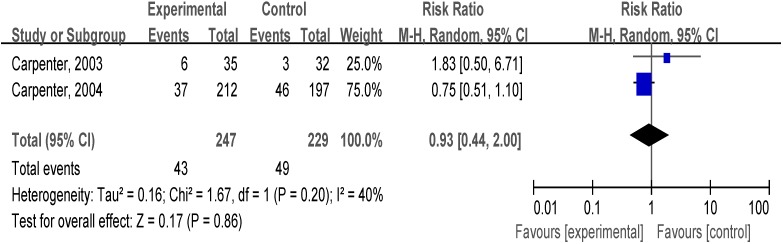
Forest plot of comparison: reduction support plus medication *vs.* other support plus medication.

We identified two trials that examined comparisons between reduction support plus medication and other support plus medication. In an analysis combining two trials (Figure 4), there was no evidence of benefit from reduction support plus medication (RR, 0.93; 95% CI, 0.44–2.00; I^2^ = 40%).

#### 3.4.4. Reduction Support *vs.* No Intervention

One study evaluated the effect of reduction support alone. Glasgow *et al*. reported that behavioural reduction support did not significantly increase smoking abstinence compared to no intervention (RR, 1.49; 95% CI, 0.56–3.93) [26].

### 3.5. Secondary Outcomes

**Figure 5:**
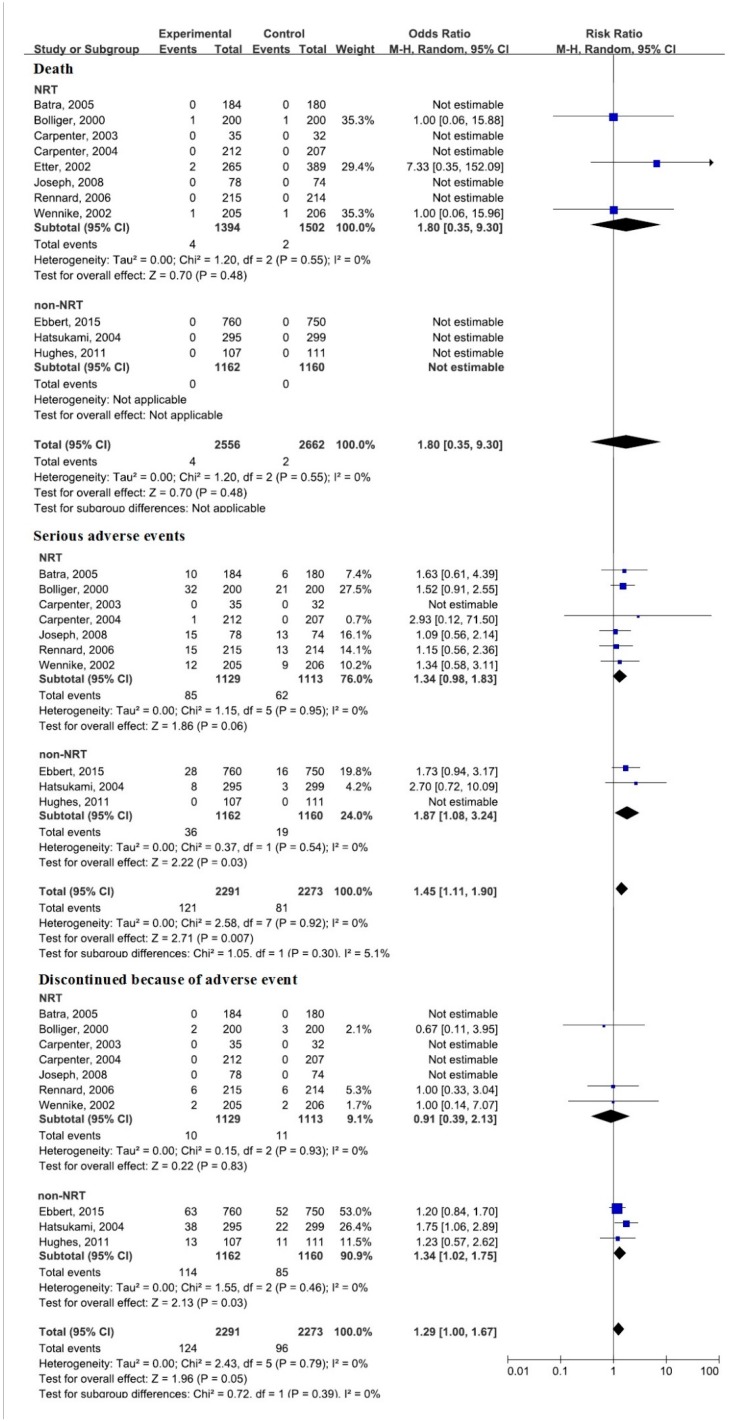
Meta-analysis of the safety outcomes.

The secondary outcome of the current study was to compare adverse events (Figure 5). Overall, 11 of the included trials reported information on adverse events (one trial only reported information on deaths). Four deaths occurred in those randomized to NRT, and no deaths occurred in those randomized to non-NRT (varenicline and bupropion). There were no significant differences between treatment and control groups (RR, 1.80; 95% CI, 0.35–9.30) in death occurrence. Serious adverse events occurred in fewer than 8% of cases in both groups. No trials reported that serious adverse events were likely to have arisen from treatment. Discontinuation because of adverse events (RR, 1.34; 95% CI, 1.02–1.75) was significantly more common for the non-NRT group, which experienced more serious adverse events (RR, 1.87; 95% CI, 1.08–3.24), compared with the control group.

### 3.6. Sensitivity Analysis

Because of the small numbers of studies for comparisons, sensitivity analyses were performed for two comparisons: reduction support plus medication versus reduction support plus placebo and reduction support plus medication versus no intervention. Further exclusion of any single trial did not significantly alter the overall combined RR, which ranged from 1.78 (95% CI, 1.18–2.67) to 2.38 (95% CI, 1.46–3.88) and 1.53 (95% CI, 1.07–2.19) to 2.11 (95% CI, 1.50–2.97).

In addition, sensitivity analyses were performed to explore the influences of various exclusion criteria (Supplementary Table S1). If we only included the trials that had large sample sizes, were performed at multiple centres, included healthy populations, and reported carbon monoxide-confirmed quit rates, the results did not significantly affect the overall pooled estimate.

### 3.7. Publication Bias

Publication bias was assessed using Egger and Begg tests. For smoking reduction plus medication versus smoking reduction plus placebo, there was no potential publication bias among the nine included trials (Egger’s test, *p* = 0.532; Begg’s test, *p* = 0.490). Publication bias was not assessed for other comparisons, because the low power with less than five trials limited the interpretability of the finding.

## 4. Discussion

The present systematic review and meta-analysis identified 14 trials (a total of 7981 smokers) investigating the effects of different types of reduction therapies for assisting smoking reduction to achieve the goal of long-term cessation in smokers without an intention to quit. The results suggested that reduction support in combination with smoking cessation medication (varenicline or NRT) significantly increased the cessation rate among smokers without an intention to quit. Moreover, the percentages of smokers with serious adverse events were not significantly different between the treatment and control (NRT) groups. Measurements of varenicline and bupropion safety should be further explored. Insufficient evidence is available to test the efficacy of bupropion plus reduction support in promoting long-term cessation in this population.

According to the present meta-analysis, the combination of reduction support with varenicline significantly increased long-term and CO-confirmed (confirmed by exhaled carbon monoxide) smoking cessation rates by a factor of 2.66. The results suggested that varenicline is an effective treatment option for smokers without an intention to quit. The percentage of smokers with serious adverse events and discontinuation because of adverse events in the varenicline group was significantly different from the control group. However, because of the limited number of studies included in the current meta-analysis, further safety measurements are needed. Bupropion significantly increased short-term abstinence rates, but the rates were not sustained after bupropion was discontinued. [27] Future studies should evaluate the efficacy and safety of bupropion with a larger sample size.

We found that NRT was the most widely used reduction therapy for smokers with no intention to quit. Compared to placebo or no intervention, NRT significantly increased long-term cessation. Various inclusion criteria did not significantly affect the results. Regarding the effect of self-help reduction materials, we could not make a definitive conclusion because of the limited sample size [23,24]. Further trials should use stricter outcomes (sustained abstinence) rather than specific time points of abstinence in the future. In accordance with the previous meta-analyses, we confirmed that NRT-assisted reduction to stop smoking was an effective and safe strategy [8,9]. In a trial including patients with heart disease, Joseph et al. reported that unserious and serious adverse events were roughly distributed in the examined treatment groups [29]. Because concerns about cardiovascular and neuropsychiatric adverse events are a relatively new issue [32,33], many trials have not reported on cardiovascular and neuropsychiatric outcomes. Mathew et al. suggested that for patients receiving NRT who continued to smoke, the sympathetic nervous system might be stimulated by high nicotine serum concentrations [34]. More evidence is needed to assess the cardiovascular and neuropsychiatric events that occur during pharmacology-assisted reduction among smokers without an intention to quit.

In the current meta-analysis, we found that the RRs for NRT versus placebo with reduction support were similar to that for NRT versus no support. This result suggests that reduction support is not necessary to achieve the effect of NRT. However, due to the limited number trials (only three trials were relative studies) included in the meta-analysis, strong and definitive recommendations cannot be made on the effect of reduction support [20,21,26]. It remains unclear whether the combination of reduction support and medication to increase reduction was better than other support plus medication. Furthermore, we still cannot make a conclusion about the efficacy of behavioural reduction support alone. To the best of our knowledge, there are at least three on-going RCTs on this topic (NCT02337400, NCT02370147, and NCT 01866722). In smokers with no immediate intention to quit, the results of these trials could provide further evidence of the efficacy of reduction support.

Our analysis did not include three pilot randomized trials because of their short follow-up periods (less than 6 months) [35,36,37]. These trials used new-style tobacco products (smokeless tobacco and very low-nicotine content cigarettes) as a substitute for smoking for smokers who were not interested in quitting. It is worth noting that the efficacy and safety of these new-style tobacco products assisted in smoking reduction and future long-term cessation.

The current meta-analysis has limitations. First, various definitions and time frames were used to define an intention to quit in the included trials. Future studies should follow the common definition of a “stage-of-change” model for smokers with no intention to quit [38,39]. Second, the treatment duration (ranging from two months to 18 months) and follow-up time (ranging from six months to 60 months) varied between the trials. The longer treatment duration might achieve more effective outcomes. We used the longest follow-up time of smoking abstinence, as used previously [40,41]. Third, most of the included trials reported the point prevalence rather than the sustained abstinence rates. The effectiveness might be overvalued because of the lack of sustained abstinence. Fourth, several trials did not report the frequency or intensity of baseline and follow-up behavioural counselling. It was reported that a more intensive counselling intervention was more effective than a less intensive one [42]. Further RCTs should report outcomes with details of baseline and follow-up interventions. Finally, 11 of the included trials recruited moderate or heavy smokers (at least 10 cigarettes per day). It remains unclear whether smoking reduction would be effective for lighter smokers.

## 5. Conclusions

In summary, the evidence suggests that a combination of reduction support and medication (NRT and varenicline) to increase reduction in achieving complete cessation is effective for smokers without an intention to quit. Further evidence is needed to assess the safety of varenicline and bupropion as well as the efficacy of reduction behavioural support and bupropion in reducing the daily consumption of cigarettes with the eventual goal of quitting smoking.

## Supplementary Files

Supplementary File 1

## Abbreviations

CENTRAL: Cochrane Central Register of Controlled Trials
RCT: randomized controlled trial
RR: relative risk
95% CI: 95% confidence interval
NRT: nicotine replacement therapy
