# Supplementary material for: Effect of Smoking Reduction Therapy on Smoking Cessation for Smokers without an Intention to Quit: An Updated Systematic Review and Meta-Analysis of Randomized Controlled Trials
Source: Int J Environ Res Public Health. 2015 Aug 25;12(9):10235–53. doi: 10.3390/ijerph120910235 (PMC4586609; doi:10.3390/ijerph120910235)
Supplement: Supplementary File 1 [file ijerph-12-10235-s001.pdf]

Supplementary Information

Effect of Smoking Reduction Therapy on Smoking Cessation for Smokers without an Intention to Quit: An Updated Systematic Review and Meta-Analysis of Randomized Controlled Trials

---

1. Search Strategy

Source: PubMed
Searched on: April 24th, 2015
((tobacco reduction [title/abstract]) OR (cigarette* reduction [title/abstract]) OR (reduce smoking [title/abstract]) OR (smoking reduction [title/abstract])) AND ((unwilling to) OR ("not willing") OR (no inten*) OR ("not ready") OR ("not interest*") OR (uninterest*) OR (unmotivated)) AND ((randomized [tiab]) OR (trial* [tiab/ pt]) OR (randomly [tiab]) OR (controlled clinical trial [pt]))

1.1. Source: Embase

Searched on: April 24th, 2015

Search	Query
#1	‘tobacco reduction’
#2	‘cigarette reduction’
#3	‘reduce smoking’
#4	‘smoking reduction’
#5	‘unwilling to’
#6	‘not willing’
#7	‘no intention’
#8	‘not ready’
#9	‘not interested’
#10	uninterested
#11	unmotivated
#12	#1 OR #2 OR #3 OR #4
#13	#5 OR #6 OR #7 OR #8 OR #9 OR #10 OR #11
#14	#12 AND #13 AND ‘trial’

1.2. Source: Cochrane Central Register of Controlled Trials

Searched on: April 24th, 2015
("tobacco reduction" OR "cigarette* reduction" OR "reduce smoking" OR "smoking reduction") AND ("unwilling to" OR "not willing" OR "no inten*" OR "not ready" OR "not interest*" OR uninterest* OR unmotivated) AND trial*

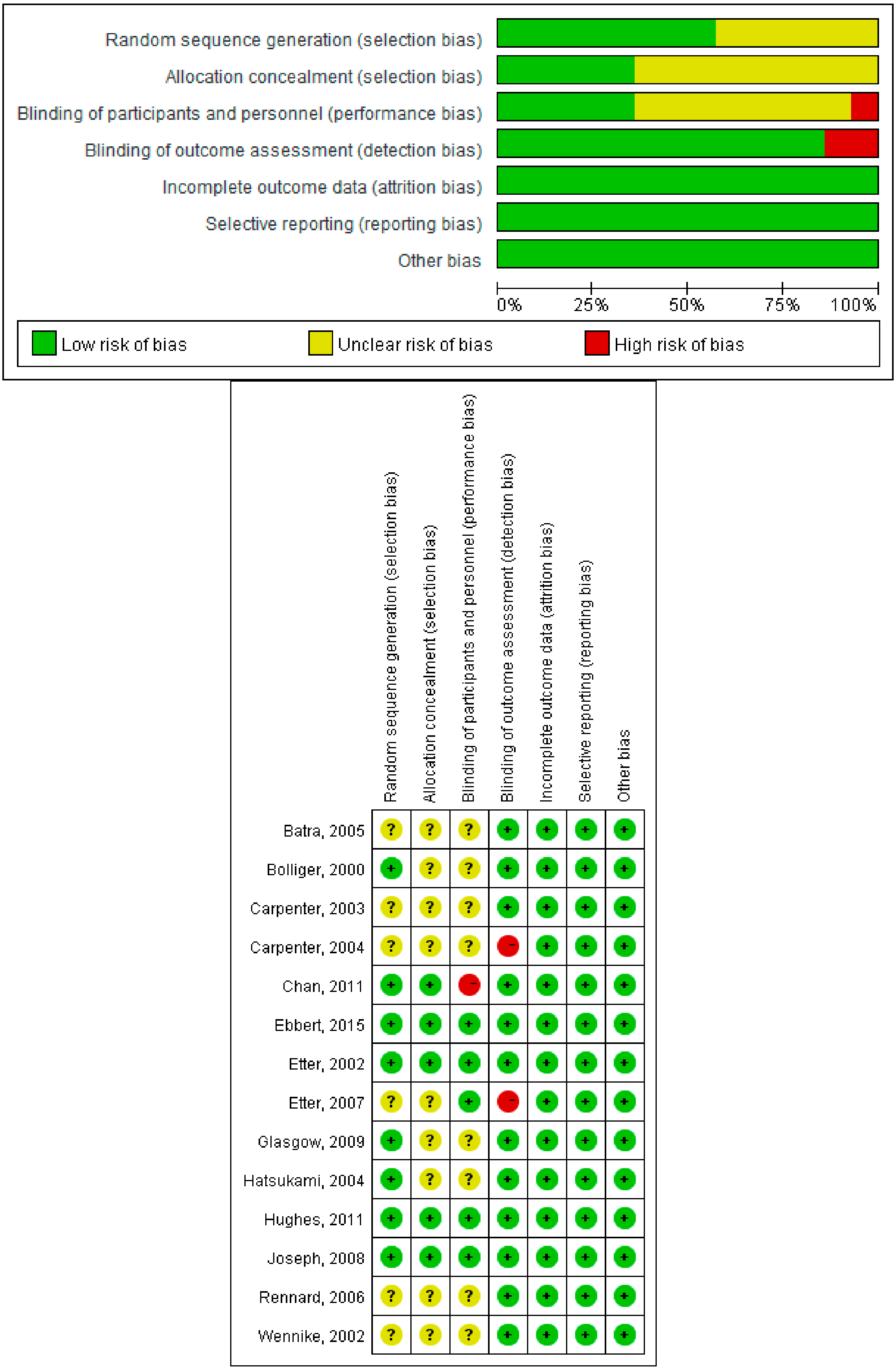

Figure S1. Risk of bias in the meta-analysis.

Table S1. Sensitivity analyses of the included trials.

Treat	No. Smokers	No. Trials	Treat /Total	Control /Total	RR (95% CI)	*p* Value	I^2, %	*p* Value for Heterogeneity
**Reduction support plus NRT *vs.* reduction support plus placebo**								
All included trials [18,19,23,24,30,31]	2356	6	111/1176	59/1180	1.94 (1.26–3.00)	0.003	45	0.11
Large sample size (n > 400) [23,24,30,31]	1592	4	70/792	35/800	2.14 (1.15–3.97)	0.02	54	0.09
Multiple center [18,19,30]	1193	3	58/599	27/594	2.40 (1.02–5.63)	0.05	66	0.01
Carbon monoxide-confirmed quit rates [18,19,30,31]	1604	4	77/804	34/800	2.39 (1.29–4.43)	0.005	53	0.09
Sustained abstinence [23,24]	752	2	34/372	25/380	1.39 (0.83–2.33)	0.21	5	0.31
Provided only self-help reduction materials [23,24]	752	2	34/372	25/380	1.39 (0.83–2.33)	0.21	5	0.31
**Reduction support plus NRT vs. no intervention**								
All included trials [21–24,29]	3033	5	150/1748	56/1285	1.93 (1.41–2.64)	<0.001	46	0.11
Large sample size (n > 400) [21–24]	2881	4	141/1670	47/1211	2.11 (1.50–2.97)	<0.001	37	0.19
Healthy population [21–24]	2881	4	141/1670	47/1211	2.11 (1.50–2.97)	<0.001	37	0.19
Carbon monoxide-confirmed quit rates [22]	1154	1	74/928	10/226	1.08 (0.95–3.43)	0.07	-	-
Sustained abstinence [23,24]	1408	2	30/530	28/778	1.57 (0.95–2.60)	0.08	0	0.94

NRT, nicotine replacement therapy.

© 2015 by the authors; licensee MDPI, Basel, Switzerland. This article is an open access article distributed under the terms and conditions of the Creative Commons Attribution license (http://creativecommons.org/licenses/by/4.0/).
